# Impact of epistasis effects on the accuracy of predicting phenotypic values of residual feed intake in U. S Holstein cows

**DOI:** 10.3389/fgene.2022.1017490

**Published:** 2022-11-01

**Authors:** Zuoxiang Liang, Dzianis Prakapenka, Kristen L. Parker Gaddis, Michael J. VandeHaar, Kent A. Weigel, Robert J. Tempelman, James E. Koltes, José Eduardo P. Santos, Heather M. White, Francisco Peñagaricano, Ransom L. Baldwin VI, Yang Da

**Affiliations:** ^1^ Department of Animal Science, University of Minnesota, Saint Paul, MN, United States; ^2^ Council on Dairy Cattle Breeding, Bowie, MD, United States; ^3^ Department of Animal Science, Michigan State University, East Lansing, MI, United States; ^4^ Department of Animal and Dairy Sciences, University of Wisconsin, Madison, WI, United States; ^5^ Department of Animal Science, Iowa State University, Ames, IA, United States; ^6^ Department of Animal Sciences, University of Florida, Gainesville, FL, United States; ^7^ Animal Genomics and Improvement Laboratory, ARS, USDA, Beltsville, MD, United States

**Keywords:** RFI, holstein, epistasis, SNP, genomic prediction, heritability, GBLUP, GREML

## Abstract

The impact of genomic epistasis effects on the accuracy of predicting the phenotypic values of residual feed intake (RFI) in U.S. Holstein cows was evaluated using 6215 Holstein cows and 78,964 SNPs. Two SNP models and seven epistasis models were initially evaluated. Heritability estimates and the accuracy of predicting the RFI phenotypic values from 10-fold cross-validation studies identified the model with SNP additive effects and additive × additive (A×A) epistasis effects (A + A×A model) to be the best prediction model. Under the A + A×A model, additive heritability was 0.141, and A×A heritability was 0.263 that consisted of 0.260 inter-chromosome A×A heritability and 0.003 intra-chromosome A×A heritability, showing that inter-chromosome A×A effects were responsible for the accuracy increases due to A×A. Under the SNP additive model (A-only model), the additive heritability was 0.171. In the 10 validation populations, the average accuracy for predicting the RFI phenotypic values was 0.246 (with range 0.197–0.333) under A + A×A model and was 0.231 (with range of 0.188–0.319) under the A-only model. The average increase in the accuracy of predicting the RFI phenotypic values by the A + A×A model over the A-only model was 6.49% (with range of 3.02–14.29%). Results in this study showed A×A epistasis effects had a positive impact on the accuracy of predicting the RFI phenotypic values when combined with additive effects in the prediction model.

## Introduction

Residual feed intake (RFI) is a measure of an animal’s feed efficiency (Koch et al., 1963; Kennedy et al., 1993; Pryce et al., 2014; Tempelman et al., 2015). In U.S. Holstein cattle, genome-wide association study (GWAS) and genomic prediction of RFI using single nucleotide polymorphism (SNP) markers have been reported (Yao et al., 2013; VanRaden et al., 2018; Li et al., 2019; Li et al., 2020). A national genomic evaluation has been implemented for feed-saved that is calculated based on RFI and body weight composite (Gaddis et al., 2021) using a SNP additive model. Additive effects are inheritable and genomic estimated breeding values (GEBV) based on additive effects are used for selecting breeding individuals. Additive effects also affect a cow’s lifetime performance. A selection index of GEBV’s of dairy traits named net merit is used as a measure of lifetime profit (VanRaden et al., 2014). Nonadditive effects including dominance and epistasis effects affect a cow’s lifetime performance if they exist but the inheritance of nonadditive effects is more complicated than that of additive effects and is discussed towards the end of this article. Given that approximately 1 million Holsteins in the U.S. were genotyped in 2020 and 2021 (CDCB, 2022) and the RFI phenotype is expensive to measure, an increase in the accuracy of predicting future RFI phenotypic performance may result in a reduction in the dairy expenses. In this study, we investigate the impact of epistasis effects on the genomic heritability of RFI and the accuracy of predicting the RFI phenotypic values of U.S. Holstein cows using prediction models with SNP additive, dominance and epistasis effects up to the third-order.

## Materials and methods

### Holstein population, SNP and phenotypic data

The Holstein population in this study had 6215 cows with RFI phenotypic observations and 78,964 imputed SNPs. The RFI phenotypic values were the phenotypic residuals after removing fixed non-genetic effects available from the December 2021 U.S. Holstein genomic evaluation data. The 6215 phenotypic values had a bell-shaped distribution with long tails. To evaluate the potential impact of the extreme phenotypic values, we evaluated the prediction accuracy after removing 17 phenotypic observations that were four standard deviations from the mean existed. The removal of 17 such extreme phenotypic values reduced the number of observations to 6198 with a similar bell-shaped distribution without the long tails of the original data (Supplementary Figure S1 and Table 1). However, the original dataset of 6215 cows had slightly higher accuracy (0.231) of predicting the phenotypic values than the dataset after removing the 17 extreme phenotypic values (0.227). Therefore, the original dataset with 6215 cows was used in this study.

**TABLE 1:** Genomic heritability estimates for the A-only and A + A×A prediction models.

Genetic effect	Full sample		10 training populations (mean ± SD)	
	A-only	A + A×A	A-only	A + A×A
Additive	0.171	0.141	0.171 ± 0.008	0.142 ± 0.007
A × A	—	0.263	—	0.252 ± 0.031
Total	0.171	0.404	0.171	0.394

SD, standard deviation.

### Mixed model for GBLUP and GREML

Genomic best linear unbiased prediction (GBLUP) of genetic values was used for calculating the accuracy of predicting the RFI phenotypic values, and genomic restricted maximum likelihood estimation (GREML) was used for estimating the heritability of each type of genetic effects using mixed models. The effect types evaluated by GBLUP and GREML included SNP additive and dominance effects, pairwise and third-order epistasis effects, and intra- and inter-chromosome additive × additive (A × A) effects. Details of those effect types and the mixed models are described in Da et al., 2022. Since the prediction model with additive and A × A effects was identified as the best prediction model in this study, only these effects are included in the description of the mixed model for GBLUP and GREML here for simplicity of notations. For the phenotypic data in this study that already removed fixed non-genetic effects from the phenotypic values, the mixed model with additive and A × A epistasis genetic values as well as the variance-covariance matrices of the model can be described as:

y=Xμ+Zg+e=Xμ+Z(a+d+aa+ad+dd)+e (1)

$$V=\text{Var}\left(y\right)=ZGZ^{\prime }+\sigma_{e}^{2}I$$ (2)

$$G=G_{a}+G_{d}+G_{\text{aa}}+G_{\text{ad}}+G_{\text{dd}}=\sigma_{\alpha }^{2}A+\sigma_{\delta }^{2}D+\sigma_{\alpha \alpha }^{2}AA+\sigma_{\alpha \delta }^{2}AD+\sigma_{\delta \delta }^{2}DD$$ (3)

where **y** = column vector of phenotypic observations, **Z** = incidence matrix allocating phenotypic observations to each individual = identity matrix for one observation per individual, µ = mean of the phenotypic values, **X** = column vector of 1’s as the model matrix for µ, **a** = column vector of genomic additive values, **d** = column vector of genomic dominance values, aa = column vector of genomic additive × additive (A×A) values, ad = column vector of genomic additive × dominance (A×D) values, dd = column vector of genomic dominance × dominance (D×D) values, **g** = column vector of total genetic values = a+d+aa+ad+dd , **e** = column vector of random residuals, $\sigma_{e}^{2}$ = residual variance, I = identity matrix, $G_{a}=\text{Var}\left(a\right)=\sigma_{\alpha }^{2}A$ , $G_{d}=\text{Var}\left(d\right)=\sigma_{\delta }^{2}D$ , $G_{\text{aa}}=\text{Var}\left(aa\right)=\sigma_{\alpha \alpha }^{2}AA$ , $G_{\text{ad}}=\text{Var}\left(ad\right)=\sigma_{\alpha \delta }^{2}AD$ , $G_{\text{dd}}=\text{Var}\left(dd\right)=\sigma_{\delta \delta }^{2}DD$ , $\sigma_{\alpha }^{2}$ = variance of additive effects, $\sigma_{\delta }^{2}$ = variance of dominance effects, $\sigma_{\alpha \alpha }^{2}$ = variance of A×A effects, $\sigma_{\alpha \delta }^{2}$ = variance of A×D effects, $\sigma_{\delta \delta }^{2}$ = variance of D×D effects, A = genomic additive relationship matrix, D = genomic dominance relationship matrix, AA = genomic A×A relationship matrix, AD = genomic A×D relationship matrix, DD = genomic D×D relationship matrix. The calculations of the genomic epistasis relationship matrices can use either the approximate method that is the genomic version of Henderson’s Hadamard products between additive and dominance relationship matrices (Henderson, 1985; Su et al., 2012; Muñoz et al., 2014; Vitezica et al., 2017) or the exact method that removes intra-locus epistasis that should not exist from the approximate method (Jiang and Reif, 2020). The EPIHAP package implements both methods (Liang et al., 2022), and this study used the exact method. The pairwise epistasis values and their genomic relationship matrices were further divided into intra- and inter-chromosome epistasis values and genomic matrices using the approach of Da et al. (2022).

### Estimation of genomic heritability

The estimation of variance components used the method of genomic restricted maximum likelihood estimation (GREML) using a combination of EM-REML and AI-REML algorithms implemented by the EPIHAP computing package (Liang et al., 2021, Liang et al., 2022). GREML estimates of variance components were used for calculating heritability estimates from the entire sample of 6215 cows and from the 10 validation populations for calculating the observed standard deviation of each genomic heritability.

### Evaluation of prediction accuracy using cross-validation

A 10-fold cross validation study was used to evaluate the accuracy of predicting the phenotypic values of each trait for each model. Individuals with phenotypic observations were randomly divided into 10 validation populations. The first nine validation populations had equal sample size of 621 cows each and the 10th population had 626 cows. In each validation population, phenotypic values were omitted when calculating GBLUP for training and validation individuals. Four measures of prediction accuracy were compared for the final prediction models, including three measures described previously (Legarra et al., 2008), denoted as ${\hat{R}}_{0p}$ , $R_{0p}$ and ${\tilde{R}}_{0}$ ; and the fourth measure of $R_{0i}$ from the EPIHAP package (Liang et al., 2021, Liang et al., 2022), where ${\hat{R}}_{0p}$ = the observed accuracy of predicting phenotypic values as the correlation between GBLUP and the phenotypic values in each validation population and then averaged over all validation populations, $R_{0p}$ = expected accuracy of predicting phenotypic values, ${\tilde{R}}_{0}$ = expected accuracy of predicting genetic values calculated from ${\hat{R}}_{0p}$ and heritability, $R_{i}$ = accuracy of predicting genetic values as the square root of the reliability for the $i^{\text{th}}$ individual that can be a training or validation individual, and subscript ‘0’ indicates validation population. The formulations of these four accuracy measures for validation populations are:

$${\hat{R}}_{0p}=\text{corr}\left({\hat{g}}_{0},\;y_{0}\right)=\left[\sum_{k=1}^{10}\text{corr}\left({\hat{g}}_{0k},\;y_{0k}\right)\right]/10$$ (4)

$$R_{0p}=R_{0}\sqrt{h^{2}}=\left[\sum_{k=1}^{10}{\left(\sum_{i=1}^{n_{0k}}R_{0i}^{k}\sqrt{h_{k}^{2}}\right)/n}_{0k}\right]/10$$ (5)

$${\tilde{R}}_{0}={\hat{R}}_{0p}/\sqrt{h^{2}}=\left[\sum_{k=1}^{10}{\hat{R}}_{0p}^{k}/\sqrt{h_{k}^{2}}\right]/10$$ (6)

$$R_{i}=\text{corr}\left({\hat{g}}_{i},\;g_{i}\right)={\left[{\left(GZ^{\prime }PZG\right)}_{\text{ii}}/G_{\text{ii}}\right]}^{1/2}\;in\;general$$ (7)

$$={\left\{{\left(\begin{array}{l} G_{\alpha }Z^{\prime }PZG_{\alpha }+G_{\alpha \alpha }Z^{\prime }PZG_{\alpha \alpha }\\ +G_{\alpha }Z^{\prime }PZG_{\alpha \alpha }+G_{\alpha \alpha }Z^{\prime }PZG_{\alpha } \end{array}\right)}_{\text{ii}}/\left[a_{\text{ii}}\sigma_{\alpha }^{2}{+\left(\text{aa}\right)}_{\text{ii}}\sigma_{\alpha \alpha }^{2}\right]\right\}}^{1/2}\;for\;A+\mathrm{A\times A}\;model$$ (8)

where ${\hat{g}}_{0}$ = GBLUP of $g_{0}$ ; $g_{0}$ = unobservable genetic values; $y_{0}$ = phenotypic observations; subscript ‘0’ denotes validation population; “corr” stands for correlation; $h^{2}$ = genomic heritability;

$R_{0}$ = average accuracy of predicting genetic values in validation populations; $R_{0i}^{k}$ = the $R_{0i}$ value of the $k^{\text{th}}$ validation population as the square root of the reliability calculated by EPIHAP (Eq. 7); $h_{k}^{2}$ = the heritability estimate of the $k^{\text{th}}$ training population; ${\hat{R}}_{0p}^{k}$ = the ${\hat{R}}_{0p}$ value of the $k^{\text{th}}$ validation population; $n_{0k}$ = the number of individuals in the $k^{\text{th}}$ validation population; ${\hat{g}}_{i}$ = GBLUP of $g_{i}$ ; $g_{i}$ = unobservable genetic value of the $i^{\text{th}}$ individual, which can be a training or validation individual, $G_{\text{ii}}$ = the $i^{\text{th}}$ diagonal element of the **G** matrix defined by Eq. 3, and $a_{\text{ii}}$ and ${\left(\text{aa}\right)}_{\text{ii}}$ are the $i^{\text{th}}$ diagonal elements of the additive and A×A relationship matrices respectively. EPIHAP (Liang et al., 2021, Liang et al., 2022) calculates the reliability ( $R_{i}^{2}$ ) for all individuals and flags a training individual as ‘T’ and a validation individual as ‘V’. In Eq. 7 and Eq. 8, $P=V^{-1}-V^{-1}X{\left(X^{\prime }V^{-1}X\right)}^{-}X^{\prime }V^{-1}$ , and in Eq. 8,

$$V=Z\left(G_{a}+G_{\text{aa}}\right)Z^{\prime }+\sigma_{e}^{2}I_{N}$$ (9)

The $R_{i}$ of Eq. 8 is for the A + A×A model. For the additive-only (A-only) model, EPIHAP automatically removes terms involving $G_{\alpha \alpha }$ from the numerator of Eq. 8 and the **V** matrix of Eq. 9, and removes ${\left(\text{aa}\right)}_{\text{ii}}\sigma_{\alpha \alpha }^{2}$ from the denominator of Eq. 8. For the A + A×A model, EPIHAP calculates three reliabilities for GBLUP of additive values, GBLUP of A×A values and GBLUP of total genetic values as the sum of additive and A×A values. The additive reliability of the A-only model was compared with the reliability of GBLUP of total genetic values of the A + A×A model.

## Results and Discussion

### Genomic heritability estimates

Heritability estimates from the full model with additive, dominance and epistasis effects up to the third-order showed that only additive effects (A), A×A and A×A×A had nonzero heritability estimates of 0.141, 0.260 and 0.005 respectively; and SNP dominance effects (D) and dominance related epistasis effects (A×D, D×D, A×A×D, A×D×D and D×D×D) all had zero heritability (Supplementary Table S2). These results showed that only A, A×A and A×A×A could contribute to the accuracy of predicting the RFI phenotypic values. However, the A + A×A + A×A×A and A + A×A models had the same prediction accuracy that was higher than the accuracy of the A-only or A×A-only model (Supplementary Table S3), indicating that A×A×A effects had no contribution to the prediction accuracy. Therefore, the A + A×A model was identified as the best epistasis model with the smallest number of effect types among the models with the highest prediction accuracy. This model was compared with the A-only model for heritability estimates and prediction accuracy to determine whether A×A benefitted the prediction of RFI phenotypic values.

For the A-only and A + A×A models, two sets of heritability estimates were calculated, one set using the full population with 6215 cows and one set using the 10 training populations each with 5594 cows for the first nine training populations and 5589 cows for the 10th training population, and the results were similar (Table 1). Partitioning the A×A effects in the A + A×A model into intra-chromosome A×A effects ( ${\text{AA}}^{\text{intra}}$ ) and inter-chromosome A×A effects ( ${\text{AA}}^{\text{inter}}$ ), the intra-chromosome A×A heritability was only 0.003, and the inter-chromosome A×A heritability was 0.260, whereas the additive heritability of 0.141 remained unchanged with or without partitioning A×A into intra- and inter-chromosome A×A effects. Under the ${\text{AA}}^{\text{intra}}$ + ${\text{AA}}^{\text{inter}}$ model, the intra-chromosome A×A heritability was 0.224, and the inter-chromosome A×A heritability was 0.289 (Supplementary Table S2). The comparison of the ${\text{AA}}^{\text{intra}}$ heritability estimates under the A + A×A and ${\text{AA}}^{\text{intra}}$ + ${\text{AA}}^{\text{inter}}$ models showed that the inter-chromosome A×A effects were responsible for the increases in prediction accuracy due to A×A effects, and intra-chromosome A×A effects were virtually completely accounted for by additive effects when additive and A×A effects were in the same model. The accuracy of predicting the RFI phenotypic values showed that the accuracies of the A + A×A and A+ ${\text{AA}}^{\text{inter}}$ models were the same (Supplementary Table S3), confirming that intra-chromosome A×A effects had no contribution to the prediction accuracy. However, A + A×A was still considered the best prediction model because no computing benefit was expected by removing ${\text{AA}}^{\text{intra}}$ from the prediction model. This was the first known study showing that additive and intra-chromosome A×A effects were virtually completely confounded whereas additive and inter-chromosome A×A effects virtually had no confounding in terms of accuracies of predicting the RFI phenotypic values and the RFI heritability estimates. Such results were consistent with the results of a Holstein epistasis GWAS that found confounding between additive and intra-chromosome A×A effects and hypothesized that genetic selection based on genome-wide SNP additive effects likely accounted for most intra-chromosome A×A effects of the five production traits (Prakapenka et al., 2021). The RFI results of this study and the epistasis GWAS results of the production traits provided accumulating evidence towards understanding the relationship between additive and intra-chromosome A×A effects.

### Accuracy of predicting RFI phenotypic values in validation populations

**FIGURE 1:**
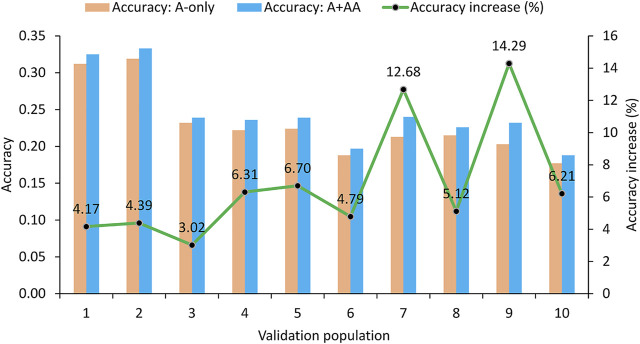
Accuracy of predicting RFI phenotypic values form 10-fold validation studies using 6215 Holstein cows.

Since the A + A×A model was identified as the best epistasis model, the evaluation of the impact of epistasis effects on the accuracy of predicting RFI phenotypic values was based on the comparison between the A-only and A + A×A models in validation populations. Results of the 10-fold validation study showed that the accuracy of predicting the RFI phenotypic values in the validation populations was 0.231 for the A-only model and was 0.246 for the best epistasis model (A + A×A), a 6.49% accuracy increase due to A×A effects over the accuracy of the A-only model (Table 2; Figure 1). In all ten validation populations, the A + A×A model was more accurate than the A-only model. The range of the prediction accuracy was 0.188–0.319 for the A-only model and was 0.197–0.333 for the A + A×A model, and the range of the increases in prediction accuracy of the A + A×A model over the A-only model was 3.02–14.29% (Figure 1). The standard deviation (SD) of the prediction accuracy was 0.045 for either model. The range of the prediction accuracy in terms of standard deviations from the mean was 0.96–1.96 SD for the A-only model and was 1.09–1.93 SD for the A + A×A model. These results showed that the variations of the two prediction models were all within the range of 1.09–1.96 SD or approximately −1.00 SD to 2.00 SD. Under the assumption of a normal distribution for the accuracy of predicting the RFI phenotypic values, the 95% interval of prediction accuracies from validations would be in the range of Mean ± 2.00 SD, and the observed ranges of 0.96–1.96 SD for the A-only model and 1.09–1.93 SD for the A + A×A model were all within the 95% interval. For the accuracy increase of the A + A×A model relative to the A-only model, the assumed 95% interval was −0.95% to 13.95%. The observed range of the accuracy increases in the ten validation populations was 3.02–14.29%, where the upper bound exceed that of the assumed 95% interval.

**TABLE 2:** Accuracy of predicting RFI phenotypic values in validation populations.

Model	Accuracy ( ${\hat{R}}_{0p}$ , mean ± SD)	Increase (%, mean ± SD)
A-only	0.231 ± 0.045	0.00 ± 0.00
A + A×A	0.246 ± 0.045	6.49 ± 3.73

SD = standard deviation. ${\hat{R}}_{0p}$ is defined by Eq. 4. ‘Increase’ = 100×[( ${\hat{R}}_{0p}$ of A + A×A model) − ( ${\hat{R}}_{0p}$ of A-only model)]/( ${\hat{R}}_{0p}$ of A-only model).

### Understanding of A×A heritability and prediction accuracy

The comparison of heritability estimates and prediction accuracy between the training and validation populations provided an understanding of the performance of A×A effects. The best prediction model for the training populations was the A×A-only model, with the highest prediction accuracy of 0.988 (Supplementary Table S3). This high prediction accuracy explained why the A×A-only model had the highest total heritability of 0.538, compared to the next highest total heritability of 0.406 from the full model with epistasis effects up to the third-order (Supplementary Table S3). This high prediction accuracy in the training populations likely was due to a mixture of model overfitting that accounted for some random residuals and true genetic effects. The evidence supporting the assumption of model overfitting was the high prediction accuracy of 0.988 in the training populations and the high heritability of 0.538 that substantially reduced the contribution of the random residuals to the phenotypic variance, although the exact amount of overfitting could not be determined. The evidence supporting the assumption of true A×A effects contributing to the 0.538 heritability estimate was the zero heritability of A×D that had twice as many effects as A×A, the zero heritability of D×D effects that had the same number of effects as the A×A, and the zero or nearly zero heritability of the third-order epistasis with many more effects than A×A (Supplementary Table S2). It is interesting to note that the integration of the A and A×A effects in the same model likely removed some inflation of the A×A heritability form the A×A-only model because the A×A heritability from the A + A×A model was 0.263, about half of the 0.538 heritability estimate from the A×A-only model. Since this 0.263 A×A heritability estimate still could be inflated for unknown reasons, this 0.263 estimate could be considered the upper bound of the true A×A heritability. The lower bound can be found based on the prediction efficiency of the additive and A×A effects. We define prediction efficiency as the ratio of the observed accuracy of predicting phenotypic values to the heritability estimate. Under this definition, the additive prediction efficiency of the A-only model was 0.231/0.171 = 1.35, and the A×A efficiency of the A×A-only model was 0.201/0.538 = 0.37. Therefore, the A×A efficiency was 27.41% of the additive efficiency. Requiring the additive efficiency for the A×A effects, the efficiency adjusted A×A heritability would be 0.201/1.35 = 0.155, which should be the lower bound of the A×A heritability because the main reason for the low A×A prediction efficiency likely was the low A×A genomic relationships between the validation and training individuals. Combining this result with the A×A heritability from the A + A×A model, the true A×A heritability should be in the range of 0.155–0.263.

The performance of the A×A-only model in the training and validation populations was drastically different. The A×A-only model had the highest prediction accuracy of 0.988 in the training populations and the lowest accuracy of 0.201 in the validation populations, an accuracy decrease of 12.99% relative the A-only model (Supplementary Table S3). The exact reasons for such a drastically different performance of the A×A-only model were unknown, but a likely reason was the low A×A genomic relationships between the training and validation populations relative to the additive genomic relationships, i.e., ${\left(\text{aa}\right)}_{\text{ij}}{=(a}_{\text{ij}}{)}^{2}$ (Cockerham, 1954; Henderson, 1985), where ${\left(\text{aa}\right)}_{\text{ij}}$ = A×A genomic relationship between the $i^{\text{th}}$ and $j^{\text{th}}$ individuals, and $a_{\text{ij}}$ = additive genomic relationship between the $i^{\text{th}}$ and $j^{\text{th}}$ individuals. Therefore, ${\left(\text{aa}\right)}_{\text{ij}}$ is lower than $a_{\text{ij}}$ by a factor of $a_{\text{ij}}$ . For the example of half-sibs, the expected relationships are $a_{\text{ij}}$ = 1/4 and ${\left(\text{aa}\right)}_{\text{ij}}$ = 1/16. Consequently, the similarity of the A×A GBLUP between a validation individual and the half-sib training individuals is only 1/4 of the similarity of the additive GBLUP. For this RFI dataset with 6215 cows, 19,310,005 pairwise relationships were possible. Of these, 31.70% additive relationships and only 0.98% of A×A relationships were >0.01, and 5.60% additive relationships were >0.05 whereas only 0.19% of the A×A relationships were >0.05 (Supplementary Table S4). As a result of the low A×A relationships, the A×A-only model should have low accuracy for predicting the RFI phenotypic values in the validation populations. The number of individuals in the training population related to a validation individual also affects the prediction accuracy of the validation individual. The reliability or the accuracy of predicting genetic values defined by Eq. 7 or 8 is affected by the relatedness between the validation individual and the training individuals and by the number of training individuals related to the validation individual. The higher the relatedness and the larger number of related training individuals, the higher the reliability of the validation individual. Therefore, an interesting question is how the two main prediction models, A-only and A + A×A, perform for different levels of reliability, or how different reliability levels affect the prediction accuracy.

### Accuracy of predicting RFI phenotypic values for different reliability levels

To study the effect of reliability levels on the accuracy of predicting phenotypic values, we compared the four measures of prediction accuracy defined by Eqs. 4–8 to provide an understanding of these measures in the 6215 Holstein cows, and then analyzed the accuracy of predicting the phenotypic values for cows with different reliability levels.

The A-only model had the highest average reliability of 0.339, followed by the 0.292 average additive reliability, 0.155 average reliability of the total genetic values, and 0.024 average reliability of A×A values under the A + A×A model (Table 3). The lower reliability of the A + A×A model was due to the larger genetic variance as the denominator of the reliability, $a_{\text{ii}}\sigma_{\alpha }^{2}{+\left(\text{aa}\right)}_{\text{ii}}\sigma_{\alpha \alpha }^{2}$ . The average denominator of the reliability was 223,521.02 for the A + A×A model and was 88,503.45 for the A-only model, i.e., the average $a_{\text{ii}}\sigma_{\alpha }^{2}{+\left(\text{aa}\right)}_{\text{ii}}\sigma_{\alpha \alpha }^{2}$ value of the A + A×A model was 2.53 times as large as the average $a_{\text{ii}}\sigma_{\alpha }^{2}$ value of the A-only model. In contrast, the numerators of the two models had a much smaller difference. The average numerator was 34,654.22 for the A + A×A model and was 30,093.47 for the A-only model, i.e., the numerator of the A + A×A model was 1.15 times as large as that of the A-only model. However, the accuracy of predicting phenotypic values is expected to involve both reliability and heritability as shown by Eq. 5, $R_{0p}=R_{0}\sqrt{h^{2}}$ , and a smaller reliability ( $R_{0}^{2}$ ) does not necessarily result in a smaller accuracy for predicting phenotypic values. This expected accuracy of predicting phenotypic values was 0.240 for the A-only model and 0.245 for the A + A×A model ( $R_{0p}$ values in Table 4), showing that the A + A×A model was expected to have a better accuracy for predicting phenotypic values than the A-only model, even though the A + A×A model had lower reliability than the A-only model. For the A-only model, the expected accuracy ( $R_{0p}$ = 0.240) was more than the observed accuracy ( ${\hat{R}}_{0p}$ = 0.231). For the A + A×A model, the expected accuracy ( $R_{0p}$ = 0.245) was almost the same as the observed ( ${\hat{R}}_{0p}$ = 0.246). The expected accuracy for predicting genetic values was lower than the observed, ${\tilde{R}}_{0}$ = 0.561 versus mean $R_{0i}$ = 0.581 for the A-only model, and was slightly higher than the observed for the A + A×A model, ${\tilde{R}}_{0}$ = 0.393 versus mean $R_{0i}$ = 0.390 (Table 4). These results showed that the low reliability of the A + A×A model relative to the A-only model was due to the large genetic variance of the A + A×A model relative to the genetic variance of the A-only model, and that the accuracy of predicting phenotypic values by one model with lower reliability can still be higher than that of a model with a higher reliability. With these results, we compared the accuracy of predicting phenotypic values for groups of individuals with different reliability levels.

**TABLE 3:** Reliability estimates of validation cows for the A-only and A + A×A prediction models from 10 validation populations.

GBLUP values	Reliability ( $R_{0i}^{2}$ ) of ‘A-only’ model		Reliability ( $R_{0i}^{2}$ ) of ‘A + A×A’ model	
	Mean ± SD	Min-max	Mean ± SD	Min-max
a	0.339 ± 0.048	0.182–0.508	0.292 ± 0.041	0.160–0.468
aa	—	-	0.024 ± 0.041	0.001–0.354
g = a + aa	—	—	0.155 ± 0.041	0.063–0.522

$R_{0i}^{2}$ is the squared value of Eq. 8 (modification is needed for the A-only model as described in the main text). SD = standard deviation. Min = minimum value. Max = maximum value.

**TABLE 4:** Comparison of four measures of prediction accuracy in validation populations and differences in the four measures between the two prediction models.

Model	${\hat{R}}_{0p}$	$R_{0p}=R_{0}\sqrt{h^{2}}$	${\tilde{R}}_{0}={\hat{R}}_{0p}/\sqrt{h^{2}}$	$R_{0}$
A-only (Mean ± SD)	0.231 ± 0.045	0.240 ± 0.080	0.561 ± 0.125	0.581 ± 0.007
A×A-only (Mean ± SD)	0.201 ± 0.039	0.156 ± 0.010	0.276 ± 0.060	0.213 ± 0.007
A + A×A (Mean ± SD)	0.246 ± 0.045	0.245 ± 0.090	0.393 ± 0.079	0.390 ± 0.011
Increase (%)	6.49	2.08	−29.95	−48.97

${\hat{R}}_{0p}$ is defined by Eq. 4. $R_{0i}$ is defined by Eq. 8 (modification is needed for the A-only model as described in the main text). SD = standard deviation. Increase = 100 × [(accuracy of A + A×A model) − (accuracy of A-only model)]/(accuracy of A-only model).

**FIGURE 2:**
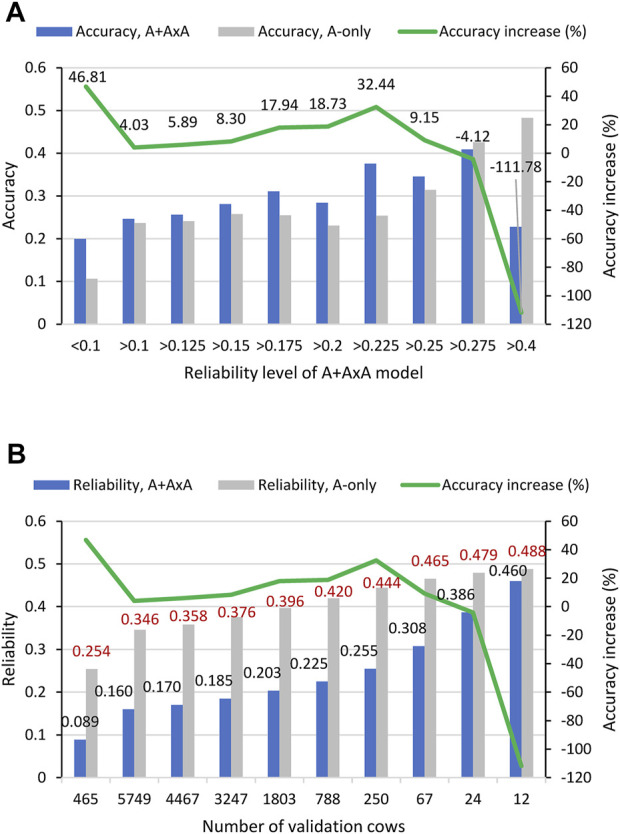
Accuracy of predicting RFI phenotypic values form 10-fold validation studies using 6215 Holstein cows for different reliability levels. **(A)** Accuracy of predicting RFI phenotypic values by the A + A×A and A-only models for different reliability levels of the GBLUP of total genetic values under the A + A×A model. The 6215 validation cows from the 10-fold validations were first sorted by the reliability of the GBLUP of the total genetic values under the A + A×A model, and the correlation between the GBLUP and phenotypic values of validation cows at each reliability level was calculated. Then the 6215 validation cows from the 10-fold validations were sorted by the reliability of the GBLUP of the additive values under the A-only model, and the correlation between the GBLUP and phenotypic values for the same number of validation cows at each reliability level of the GBLUP of total genetic values was calculated. The prediction accuracies of the two models were compared for the same number of cows. **(B)** Reliability of the GBLUP of total genetic values under the A + A×A model and additive reliability under the A-only model for the same numbers of cows. “Accuracy” is defined by Eq. 4. “Accuracy increase” = 100 × [(accuracy of A + A × A model)—(accuracy of A-only model)]/(accuracy of A-only model).

The reliability levels were determined in two ways: Sorting reliability of predicting genetic values ( $R_{0i}$ ) under the A + A×A model, and under the A-only model. Under the A + A×A model, ten levels of reliability were defined based on sorting the GBLUP reliability for total genetic values (Figure 2A). Under the A-only model, ten levels of reliability were also defined based on the sorting the GBLUP reliability of additive values such that each reliability level had the same number of cows as the corresponding reliability level under the A + A×A model (Figure 2B). The two models had the same number of cows but generally did not have the same cows at each reliability level. For example, for the highest reliability level of >0.4 with 12 cows, only two cows were common to both models. Therefore, the comparison of the ${\hat{R}}_{0p}$ values between the two models at the same reliability level generally did not compare the same cows. The same number of cows was required at each reliability level to exclude the possibility that different accuracies of the two models at the same reliability level were due to different sample sizes. The prediction accuracy ( ${\hat{R}}_{0p}$ of Eq. 4) was calculated for each reliability level and each model.

The results showed that the A + A×A model had higher accuracy of predicting the RFI phenotypic values than the A-only model for all reliability levels except the two highest reliability levels of >0.275 with 24 cows and >0.4 with 12 cows. The average reliability at each reliability of each model was 0.155–0.460 for the GBLUP of total genetic values of the A + A×A model and was 0.339–0.488 for additive reliability of the A-only model, and A-only reliability was higher than that of the A + A×A model at every reliability level (Figure 2B). For the highest reliability level with 12 cows, the A + A×A model had the worst performance relative to the A-only model, with prediction accuracy that was only 47.20% of the A-only accuracy and had lower accuracy for the second highest reliability level with 24 cows (Figure 2A). For the reliability level <0.09 with 465 cows, the A + A×A model had the highest accuracy increase relative to the A-only model, a 46.81% increase over the A-only model (Figures 2A,B). The fact that the inclusion of A×A epistasis effects in the prediction model improved the prediction accuracy over the A-only model for all cows except the 24 cows with the highest additive reliability under the A-only model indicated that the A + A×A model could be more beneficial than the A-only model for predicting the RFI phenotypic values for culling decisions, and that the A-only model could perform well for cows with the highest additive reliability. Given that RFI is expensive to measure and the growth of the RFI sample size is expected to be slow, the increase in reliability for about one million genomic evaluated cows every year is expected to be a slow process. This slow process should favor the A + A×A model for its better accuracy than the A-only model for all reliability levels except the 24 cows with the highest additive reliability.

### Negative additive relationships leading to positive A×A relationships

Pedigree additive relationship between two individuals is positive, but a genomic additive relationship can be negative and genomic A×A relationship as the product of the additive relationship with itself is positive. The negative additive relationship could be interpreted as the two individuals involved being less related than two individuals with a positive genomic relationship, but the positive A×A relationship resulting from the negative additive relationship does not have a reasonable interpretation. Although this is a problem for interpreting the A×A relationship, the negative additive relationship and the positive A×A relationship resulting from the negative additive relationship are not problems for genomic prediction, because the additive and A×A relationships originated from the model matrix of additive effects (Da et al., 2022). Modifying the additive and A×A relationships is equivalent to modifying the additive and A×A model matrices. Therefore, for genomic prediction, negative additive relationships and positive A×A relationships resulting from the negative additive relationships should be left unchanged.

For this RFI dataset with 6215 cows, 55.15% of the 19,310,005 genomic additive relationships between two cows were negative, with an average value −0.022 (Supplementary Table S4). For the general formula of A×A relationship, ${\left(\text{aa}\right)}_{\text{ij}}{=(a}_{\text{ij}}{)}^{2}$ , A×A relationship ${\left(\text{aa}\right)}_{\text{ij}}$ could not be negative even when the additive relationship $a_{\text{ij}}$ is negative. However, the genomic version of ${\left(\text{aa}\right)}_{\text{ij}}{=(a}_{\text{ij}}{)}^{2}$ contains intra-locus epistasis that should not exist (Martini et al., 2020), and hence the genomic A×A relationship matrix based on ${\left(\text{aa}\right)}_{\text{ij}}{=(a}_{\text{ij}}{)}^{2}$ is an approximate genomic epistasis relationship matrix (AGERM). This study used the exact genomic epistasis relationship matrix (EGERM) that removes the intra-locus epistasis by subtracting a quantity from ${\left(\text{aa}\right)}_{\text{ij}}{=(a}_{\text{ij}}{)}^{2}$ (Jiang and Reif, 2020), and observed negative A×A relationships that had very small absolute values. These negative A×A relationships accounted for 10.56% of the 19,310,005 pairwise A×A relationships with an average value −0.000009 ≈ 0. Therefore, the problem of negative additive relationships leading to positive A×A relationships was negligible in this RFI dataset when the EGERM method was used. Using AGERM, no negative A×A relationships were observed. However, the differences between EGERM and AGERM were negligible with differences at the third or fourth decimal point (Supplementary Table S4), and the two methods had the same heritability estimates and prediction accuracy. Therefore, EGERM and AGERM were equally good for this study in terms of heritability estimates and prediction accuracy although we chose to use EGERM.

### Inheritance of nonadditive effects

Additive effects are allelic effects and are inheritable because each parent transmits one allele of each locus to the offspring. This type of inheritance of transmitting the exact genetic material for a genetic effect from parents to offspring will be referred to as “direct inheritance”. The discussion of nonadditive inheritance uses the examples of dominance effects and A×A epistasis effects. The mating system is assumed a sire mating with many dams. With this mating system, the inheritance of dominance is indirect inheritance, whereas the inheritance of A×A effects involves both direct and indirect inheritance. We use “indirect inheritance” to refer to the inheritance where a parental allelic combination in daughters is the favorable genetic effect while each parental allele does not have a genetic effect.

Dominance effect is the difference between the heterozygous genotypic value and the average of the two homozygous genotypic values. A nonzero dominance effect is partial dominance if the heterozygous genotypic value is between two homozygous genotypic values, complete dominance if the heterozygous genotypic value is the same as one of the two homozygous genotypic values, or overdominance if the heterozygous genotypic value is more extreme than either of the two homozygous genotypic values. A genome-wide association study (GWAS) in Holstein cattle detected positive overdominance effects for the three yield traits and negative overdominance effects for fat and protein percentages (Jiang et al., 2019). The result of daughter genotypic arrays for given sire genotypes showed that the frequency of the offspring heterozygous genotype (*Aa*) was q, ½ and p for sire genotypes of *AA*, *Aa* and *aa* respectively, where p = frequency of the *A* allele and q = the frequency of the *a* allele in the population (Supplementary Table S3). Assuming p > q and q < ½, the *aa* sire genotype results in the highest heterozygosity in daughters, followed by *Aa* and *AA*. To quantify the impact of indirect inheritance, the difference between the heterozygosity of the daughters and the heterozygosity of the population will be termed as “daughter difference”, and the daughter difference relative to the population heterozygosity termed as ‘relative daughter difference’ (λ, Supplementary Table S5). Homozygous sire genotype of a rare allele (*A* or *a*) has the largest impact on increasing the heterozygosity in daughters. Based on this analysis, selection for sires with high dominance values in daughters theoretically could be selecting sires with the homozygous genotype of a rare allele and could dramatically increase the heterozygosity in future daughters and improve the dominance values of the future population, if the heterozygous genotype has a beneficial overdominance effect. As allele frequencies become closer to be equal, indirect inheritance diminishes, and dominance effect becomes completely noninheritable (Supplementary Figure S2).

An A×A effect is the interaction effect between the additive effects of two loci. Assuming two biallelic loci with alleles *A* and *a* at locus 1 and alleles *B* and *b* at locus 2, four allelic combinations between the two loci are possible, *AB*, *Ab*, *aB*, and *ab*; and nine two-locus genotypes are possible, *AABB*, *AABb*, *AAbb*, *AaBB*, *AaBb*, *Aabb*, *aaBB*, *aaBb*, and *aabb*. Assuming linkage equilibrium and the *AB* allelic combination to be the favorable combination and the other three combinations to be the unfavorable combinations, the inheritance of the favorable allelic combination (*AB*) is direct heritance for one sire genotype (*AABB*), is indirect inheritance for four sire genotypes (*Aabb*, *AAbb*, *aaBB aaBb*), and is a mixture of direct and indirect inheritance for two genotypes (*AABb*, *AaBB*), whereas the doubly heterozygous genotype (*AaBb*) does not change the gametic or genotypic frequencies of the daughters, and the inheritance of the *ab* combination is direct inheritance for the *aabb* sire genotype. This analysis shows that the inheritance of A×A effects involves both direct and indirect inheritance and is much more complex than that of additive effect. For this reason, the value of including A×A effect for sire selection should require further study. For predicting phenotypic values, the issue of A×A inheritance is not involved. Therefore, the utility of including A×A in the prediction model currently is for predicting phenotypic values that should benefit cow culling decision with respect to the cow’s phenotypic potential.

## Conclusion

The inclusion of additive × additive effects along with additive effects in the prediction model improved the accuracy of predicting the RFI phenotypic values in U.S. Holstein cows over the accuracy of the additive-only model and is expected to have a positive economic impact on selecting the top 30% of cows with the best genomic evaluations. The nearly complete confounding between additive and intra-chromosome A×A effects and the nearly complete absence of confounding between additive and inter-chromosome A×A effects in terms of prediction accuracy and heritability estimates provided new evidence towards understanding the relationship between additive and A×A effects.

## Data Availability

Feed efficiency data are available to the public in summarized form, as predicted breeding values for residual feed intake and feed saved. Access to the original phenotypes for dry matter intake, secreted milk energy, metabolic body weight, body weight change, and residual feed intake may be requested, for research purposes, by contacting university partners in the multi-state feed efficiency collaborative. (Contact: Michael VandeHaar, mikevh@msu.edu).
