# Supplementary material for: Impact of epistasis effects on the accuracy of predicting phenotypic values of residual feed intake in U. S Holstein cows
Source: Front Genet. 2022 Nov 1;13:1017490. doi: 10.3389/fgene.2022.1017490 (PMC9664219; doi:10.3389/fgene.2022.1017490)
Supplement: Supplementary file 1 [file DataSheet1.PDF]

# Supplementary Material

## Impact of epistasis effects on the accuracy of predicting phenotypic values of residual feed intake in U.S. Holstein cows

**TABLE S1 |** Basic statistics of residual feed intake (RFI) phenotypic values (kg).

| | N | Mean | SD | Min | Max |
|---|---|---|---|---|---|
| Original data | 6215 | −54.62 | 330.18 | −1955.34 | 1798.91 |
| Outliers removed | 6198 | −56.70 | 320.66 | −1349.94 | 1250.68 |

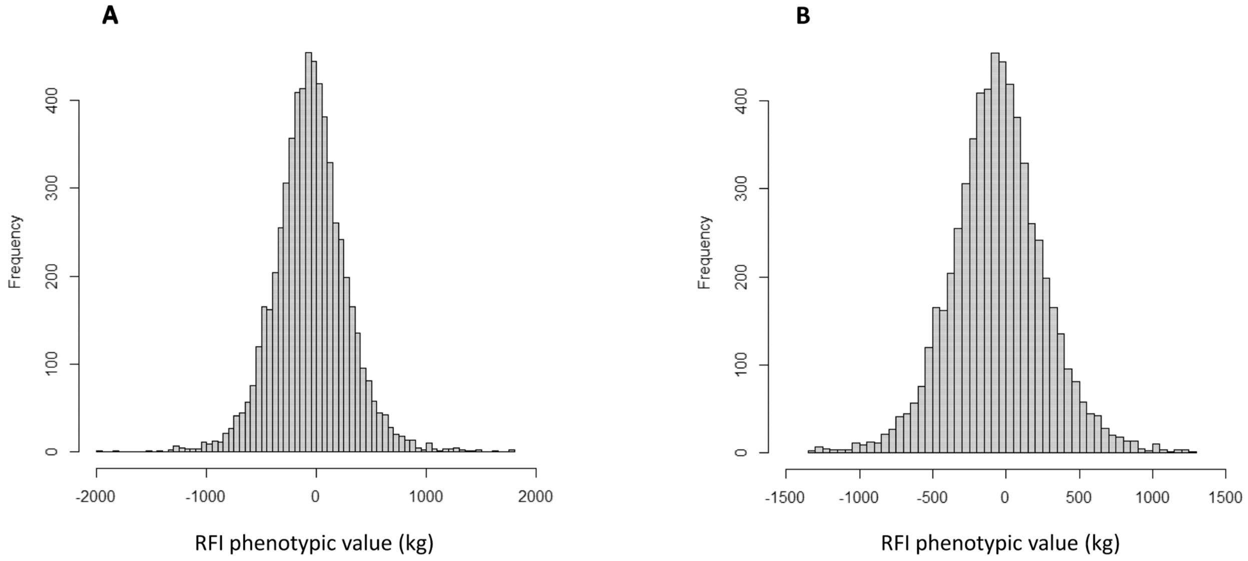


**FIGURE S1 |** Distribution of residual feed intake (RFI) phenotypic values. (**A**) Original dataset of 6215 cows. (**B**) Edited dataset of 6198 cows after removing 17 outliers with four standard deviations or more from the mean.

**TABLE S2 |** Genomic heritability estimates for nine prediction models.

| Heritability | Model | | | | | | | | |
|---|---|---|---|---|---|---|---|---|---|
| | A | A+D | AA | $AA^{intra}$ $+AA^{inter}$ | A+AA | $A+AA^{intra}$ $+AA^{inter}$ | $A+AA^{inter}$ | A+D+AA +AD+DD | A+D+AA +AD+DD +AAA+AAD +ADD+DDD |
| A | 0.171 | 0.171 | - | - | 0.141 | 0.141 | 0.141 | 0.141 | 0.141 |
| D | - | 0.000 | - | - | - | - | - | 0.000 | 0.000 |
| AA | - | - | 0.538 | - | 0.263 | - | - | 0.263 | 0.260 |
| $AA^{intra}$ | - | - | - | 0.224 | - | 0.003 | - | - | - |
| $AA^{inter}$ | - | - | - | 0.289 | - | 0.260 | 0.263 | - | - |
| AD | - | - | - | - | - | - | - | 0.000 | 0.000 |
| DD | - | - | - | - | - | - | - | 0.000 | 0.000 |
| AAA | - | - | - | - | - | - | - | - | 0.005 |
| AAD | - | - | - | - | - | - | - | - | 0.000 |
| ADD | - | - | - | - | - | - | - | - | 0.000 |
| DDD | - | - | - | - | - | - | - | - | 0.000 |
| Total | 0.171 | 0.171 | 0.538 | 0.512 | 0.404 | 0.404 | 0.404 | 0.404 | 0.406 |

A = additive effects, D = dominance effects, AA = A×A epistasis effects, AD = A×D epistasis effects, DD = D×D epistasis effects, AAA = A×A×A epistasis effects, AAD = A×A×D epistasis effects, ADD = A×D×D epistasis effects, DDD = D×D×D epistasis effects.

**TABLE S3 |** Accuracy of predicting RFI phenotypic values in training (T) and validation (V) populations for nine prediction models.

| Model | Accuracy | | Increase (%) | |
|---|---|---|---|---|
| | T | V | T | V |
| A | 0.644 | 0.231 | 0.00 | 0.00 |
| A+D | 0.644 | 0.231 | 0.00 | 0.00 |
| AA | 0.988 | 0.201 | 53.42 | -12.99 |
| $AA^{intra}$ $+AA^{inter}$ | 0.983 | 0.202 | 52.64 | -12.55 |
| A+AA | 0.932 | 0.246 | 44.72 | 6.49 |
| $A+AA^{intra}$ $+AA^{inter}$ | 0.932 | 0.245 | 44.72 | 6.06 |
| $A+AA^{inter}$ | 0.932 | 0.246 | 44.72 | 6.49 |
| A+D+AA+AD+DD | 0.933 | 0.245 | 44.88 | 6.06 |
| A+D+AA+AD+DD+AAA+ADD+AAD+DDD | 0.935 | 0.246 | 45.03 | 6.49 |

'Accuracy' is defined by Equation 4 in the main text. 'Increase' is the accuracy increase of the epistasis model over the accuracy of A-only model for predicting the RFI phenotypic values, i.e., Increase = 100×[(accuracy of epistasis model) − (accuracy of A-only model)]/ (accuracy of A-only model).

**TABLE S4 |** Examples of genomic additive and A×A relationships among 6215 cows with total 19,310,005 pairwise relationships.

| Genomic relationship | <0 | | >0.01 | | >0.05 | |
|---|---|---|---|---|---|---|
| | % | Min,<br>Mean,<br>Max | % | Min,<br>Mean,<br>Max | % | Min,<br>Mean,<br>Max |
| Additive | 55.15 | -0.132288,<br>-0.0217115,<br>-0.0000000156751 | 31.70 | 0.0100001,<br>0.0352598,<br>1.04758 | 5.60 | 0.0500001,<br>0.08286564,<br>1.04758 |
| A×A (EGERM) | 10.56 | -0.0000189288,<br>-0.0000092992,<br>-3($10^{-11)}$ | 0.98 | 0.0100001,<br>0.0315942,<br>1.09626 | 0.19 | 0.0500005,<br>0.08456942,<br>1.09626 |
| A×A (AGERM) | 0 | Not available | 0.98 | 0.0100001,<br>0.03157935,<br>1.09741 | 0.190378 | 0.0500003,<br>0.0846079,<br>1.09741 |

**TABLE S5 |** Indirect inheritance of sire dominance effects as a change of the heterozygosity in daughters relative to the population heterozygosity.

| Sire genotype | *AA* | *Aa* | *aa* |
|---|---|---|---|
| Sire gametic array | 100% *A* | 0.5*A*+0.5*a* | 100% *a* |
| Dam gametic array | p*A* + q*a* | p*A* + q*a* | p*A* + q*a* |
| Offspring genotypic array | p(*AA*)+ q(*Aa*) | 0.5[ p(*AA*)+ q(*Aa*)] +<br>0.5[ p(*Aa*)+ q(*aa*)] | p(*Aa*)+ q(*aa*) |
| Frequency of *Aa* in daughters | q | 0.5 | p |
| Difference between daughters and population (daughter difference) | $q-2pq$ | $0.5-2pq$ | $p-2pq$ |
| Relative daughter difference | $(q-2pq)/2pq$<br>$=1/(2p)-1$ | $(0.5-2pq)/2pq$<br>$=1/(4pq)-1$ | $(p-2pq)/2pq$<br>$=1/(2q)-1$ |

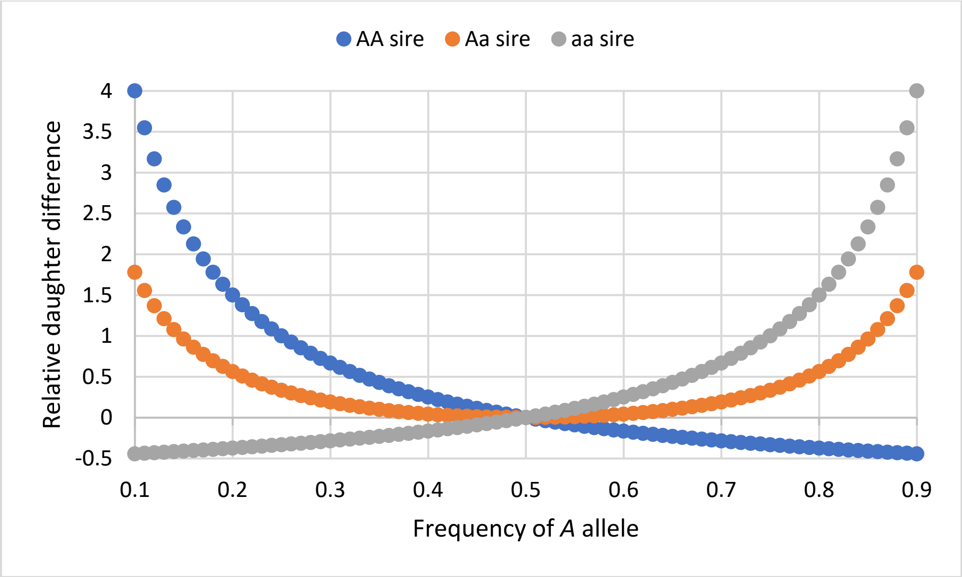


**FIGURE S2 |** Indirect inheritance of sire dominance effects as a function of allele frequencies in the population. Homozygous sire genotype of a rare allele has the largest impact on increasing the heterozygosity in daughters. As allele frequencies become closer to equal frequencies, indirect inheritance diminishes, and sire dominance effect becomes completely noninheritable.
